# Curcumin enhances p-cresyl sulfate-induced cytotoxic effects on renal tubular cells

**DOI:** 10.7150/ijms.72646

**Published:** 2022-06-27

**Authors:** Chyou-Wei Wei, Tsai-Kun Wu, Shu-Cing Wu, Yi-Lin Chen, Ying-Ru Pan, Yi-Chung Chien, Jia-Yan Wu, Yung‑Lung Yu, Giou-Teng Yiang

**Affiliations:** 1 Department of Nutrition, Master Program of Biomedical Nutrition, Hungkuang University, Taichung 43302, Taiwan.; 2 Division of Renal Medicine, Tungs' Taichung MetroHarbor Hospital, Taichung 43503, Taiwan.; 3 College of Medicine, National Chung Hsing University, Taichung 40227, Taiwan.; 4 Department of Biotechnology and Animal Science, National Ilan University, Yilan 26407, Taiwan.; 5 Program of Indigenous Education in College of Bioresources, National Ilan University, Yilan, 26407 Taiwan.; 6 Department of Medical Research, Tungs' Taichung Metroharbor Hospital, Taichung, Taiwan.; 7 Graduate Institute of Biomedical Sciences, China Medical University, Taichung 40402, Taiwan.; 8 Institute of Translational Medicine and New Drug Development, China Medical University, Taichung 40402, Taiwan.; 9 Drug Development Center, Research Center for Cancer Biology, China Medical University, Taichung 40402, Taiwan.; 10 Center for Molecular Medicine, China Medical University Hospital, Taichung 40402, Taiwan.; 11 Department of Medical Laboratory Science and Biotechnology, Asia University, Taichung 41354, Taiwan.; 12 Department of Emergency Medicine, Taipei Tzu Chi Hospital, Buddhist Tzu Chi Medical Foundation, New Taipei 23142, Taiwan.; 13 Department of Emergency Medicine, School of Medicine, Tzu Chi University, Hualien 97002, Taiwan.

**Keywords:** Indoxyl sulfate, p-cresyl sulfate, Curcumin

## Abstract

Indoxyl sulfate (IS) and p-cresyl sulfate (PCS), protein-bound uremic toxins, can induce oxidative stress and cause renal disease progression. However, the different cytotoxic effects on renal cells between IS and PCS are not stated. Due to uremic toxins are generally found in CKD patients, the mechanisms of uremic toxins-induced renal injury are required to study. Curcumin has anti-oxidant, anti-inflammatory and anti-apoptotic effects which may be potential used to protect against renal damage. In contrast, curcumin also exert cytotoxic effects on various cells. In addition, curcumin may reduce or enhance cytotoxicity combined with different chemicals treatments. However, whether curcumin may influence uremic toxins-induced renal injury is unclear. The goal of this study is to compare the different cytotoxic effects on renal cells between IS and PCS treatment, as well as the synergistic or antagonistic effects by combination treatments with curcumin and PCS. Our experimental result shows the PCS exerts a stronger antiproliferative effect on renal tubular cells than IS treatment. In addition, our study firstly demonstrates that curcumin enhances PCS-induced cell cytotoxicity through caspase-dependent apoptotic pathway and cell cycle alteration.

## Introduction

The uremic toxins can be produced from food digestion and metabolism 1. The accumulation of uremic toxins can worsen kidney function in chronic kidney disease patients 2, 3. Indoxyl sulfate (IS) and p-cresyl sulfate (PCS), protein-bound organic compounds, are the most well-known uremic toxins in the world and both of them are not removed efficiently by hemodialysis 4, 5. The uremic toxins can induce oxidative stress and inflammation resulting in cell senescence and death on renal tubular epithelial cells 6-8. However, the mechanisms of uremic toxins-induced cytotoxicity and the different cytotoxic effects of IS and PCS remain to be studied.

Many studies have demonstrated protein-bound uremic toxins such as IS and PCS can induce reactive oxygen species (ROS) production resulting in oxidative stress increase, and cause renal disease progression and vascular disease 9-12. Due to protein-bound uremic toxins are not clearance effectively by hemodialysis, AST-120 is used to scavenge the uremic toxins currently 11, 13, 14. Previous studies showed IS can promote the epithelial-to-mesenchymal transition (EMT) and apoptosis of renal tubular cells as well as to accelerate renal disease progression 13-15. In addition, expression of ICAM-1, and activation of NF-κB, p53, and MAPK (JNK and p38) are involved on IS-induced cytotoxicity 12, 16-20. Currently, IS has been studied extensively among these protein-bound uremic toxins, however, other protein-bound uremic toxins are less studied to state 21. A study compared the similarities and differences of cytotoxic effects on pig LLC-PK1 cells, a pig porcine renal tubular epithelial cell line, among the protein-bound uremic toxins containing IS, p-cresyl sulfate (PCS), phenyl sulfate (PhS), hippuric acid (HA), and indoleacetic acid (IAA), and the study indicated PCS- and PhS-induced cytotoxic effects are similar to IS while HA and IAA are different 21. On the other hand, some papers reported PCS can induce pro-apoptotic and pro-inflammatory effect, as well as induce cell death by increasing Bax/Bcl-2 ratio, cleaved caspase-3 and Beclin-1 22, 23. However, many mechanisms of PCS-induced cytotoxic effects are remained to study. Therefore, the PCS- and IS-induced cytotoxic effects on renal tubular cells were further determined in this study.

Curcumin can exert anti-oxidant, anti-inflammatory and anti-apoptotic effects, therefore, it may be a potential material applied on various diseases including neuronal, cardiovascular, and renal disease 24-26. Previous studies showed curcumin can protect renal tubular cells against ischemia reperfusion-induced injury, cisplatin-induced nephrotoxicity, ferroptosis-mediated cell death, high glucose-induced EMT, lipopolysaccharide-induced renal inflammation, cyclosporine A‑induced fibrosis and gentamicin-induced nephrotoxicity 27-34. However, whether curcumin can reduce uremic toxins-induced cytotoxic effects on renal tubular cells is remained unclear. On the other hand, many studies showed that curcumin can cause growth arrest and apoptosis in various cells 35-38. In addition, previous studies indicated curcumin promotes cisplatin-induced cytotoxic effects 39 while curcumin reduces 5-Fluorouracil-iduced cytotoxicity 40. Taken together, these studies indicated curcumin may play virous roles to influence cell growth under different conditions. Therefore, besides to determine the PCS-induced cytotoxic effects, the aim of our study also to determine how curcumin influence PCS-induced cytotoxic effects on renal cells.

## Materials and methods

### Materials and chemicals

Indoxyl sulfate (IS), p-Cresol (PCS), cucumin and tubulin polyclonal antibody were obtained from Sigma-Aldrich (St. Louis, MO, USA). MTT assay kit was bought from Bio-basic Canada Inc (Markham, OT, Canada). Anti-cleaved PARP (1:2000; cat. no. 9544) and anti-caspase-3 (1:1000; cat. no. 9965) primary rabbit polyclonal antibodies and horseradish peroxidase (HRP)-conjugated goat anti-rabbit IgG secondary antibody (1:2,000, cat. no. 7074) were obtained from Cell Signaling Technology (Danvers, MA, USA). Western Lightning Chemiluminescence Reagent Plus was bought from Perkin Elmer (Waltham, MA, USA). Fetal bovine serum, Dulbecco's Modified Eagle Medium (DMEM), non-essential amino acid, L-glutamine, and penicillin/streptomycin were bought from GIBCO BRL (Invitrogen Life Technologies, Carlsbad, CA, USA).

### Cell lines and cell culture

Rat renal tubular epithelial cells (NRK-52E) and human renal tubular epithelial cells were obtained from the Bioresource Collection and Research Center (Shin Chu, Taiwan). Cells were maintained and cultured with DMEM medium (containing 10% fetal bovine serum, 2 mM l glutamine, 100 IU/ml penicillin/streptomycin and 0.1 mM non-essential amino acids) at 37 °C in a humidified atmosphere containing 5% CO_2_.

### Cell survival rate assay

NRK-52E and HK-2 cells were cultured at 96-well dish (1×10^4^ cells/well). Every 24 hour, the MTT assay kit was added into the control and experimental groups. After incubation at 37 °C for 3 hours, the purple formazan products were measured at 570 nm (A570) using a Multiskan™ FC Microplate Photometer (Molecular Devices, Sunnyvale, CA, USA). The cell viability (%) was indicated as (A570 experimental group)/(A570 control group) × 100%.

### Cell cycle analysis

Cell cycle was determined by using fluorescence‑activated cell sorting. The cells obtained from control and experimental groups were treated with PBS buffer and fixed with 70% alcohol at 4 °C for 1 h. Next, cells were washed with PBS for 5 min and treated with 1 ml propidium iodide (PI) solution (containing 50 μg/ml PI, 100 μg/ml RNase A, and 0.1% Triton X‑100) for 30 min at 37 °C. After washed with PBS, the cells were determined by flow cytometry (Partec CyFlow® SL; SysmexPartec GmbH, Görlitz, Germany) and analyzed with CellQuest software (Becton-Dickinson).

### Western blotting

Cells were treated with the RIPA buffer (EMD Millipore, Billerica, MA, USA). Cellular proteins were collected from the supernatant with centrifugation (16,000 × g) at 4 °C for 20 min. The protein concentration was determined by using a protein assay kit (Thermo Fischer Scientific, Inc., Waltham, MA, USA). Equal quantities (40 μg) of protein were separated by 13.3% SDS-PAGE and transferred onto PVDF membranes (EMD Millipore). The membranes were treated with 5% non-fat milk at room temperature for 2 hours and washed with PBS buffer for 15 minutes (three times). The PVDF membranes were treated with primary antibodies at room temperature for 4 hours. Next, PVDF membranes were washed three times with PBS buffer for 15 minutes, then the membranes were treated with anti-rabbit HRP-conjugated secondary antibodies at room temperature for 1 hour. Finally, the membranes were treated with Western Lightning® Chemiluminescence Plus reagent (PerkinElmer, Inc., Waltham, MA, USA) and observed with a Luminescence Image Analysis system (LAS-4000, FUJIFILM Electronic Materials Taiwan Co., Ltd., Tainan, Taiwan).

### Data Analysis

All data were collected from four independent experiments and the data were indicated as the mean ± SEM.

## Results

### PCS exerted a stronger cytotoxicity effect than IS on rat renal tubular epithelial cell line

**Figure 1:**
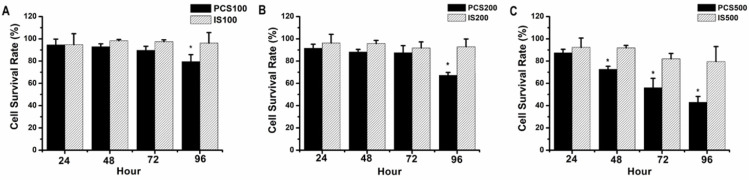
** Cell survival rate of NRK-52E cells by PCS and IS treatments. (A)** Cells were treated with 100 µM PCS or 100 µM IS. **(B)** Cells were treated with 200 µM PCS or 200 µM IS **(C)** Cells were treated with 500 µM PCS or 500 µM IS. Cell survival rates were determined at 24-96 hours by MTT assay and calculated as A570 experimental group/A570 control group × 100%. The data were calculated from four independent experiments and presented as mean ± SD. *P < 0.05.

NRK-52E, normal rat renal proximal tubular epithelial cell line, were treated with uremic toxins (PCS and IS). Our data showed that the survival rates were above 90% on 100 µM IS-treated NRK-52 cells during 24-96 hours, and the survival rates were about 90-80% on 100 µM PCS-treated NRK-52 cells during 24-96 hours (Fig. 1A). As similar result to Figure 1A, the survival rates were above 90% on 200 µM IS-treated NRK-52 cells during 24-96 hours (Fig. 1B) while the survival rate was below 70% on 200 µM PCS-treated NRK-52 cells after 96 hours (Fig. 1B). Observed on Figure 1C, the survival rates were about 90-80% on 500 µM IS-treated NRK-52 cells during 24-96 hours. However, the survival rates were 90 to 40% on 500 µM PCS-treated NRK-52 cells during 24-96 hours respectively. Taken together, our primary experiments indicated PCS exerted a stronger cytotoxicity effect than IS on rat renal tubular epithelial cell.

### Cell survival rate of human renal tubular epithelial cell line was decreased by PCS treatment with dose- and time-dependent manners

**Figure 2:**
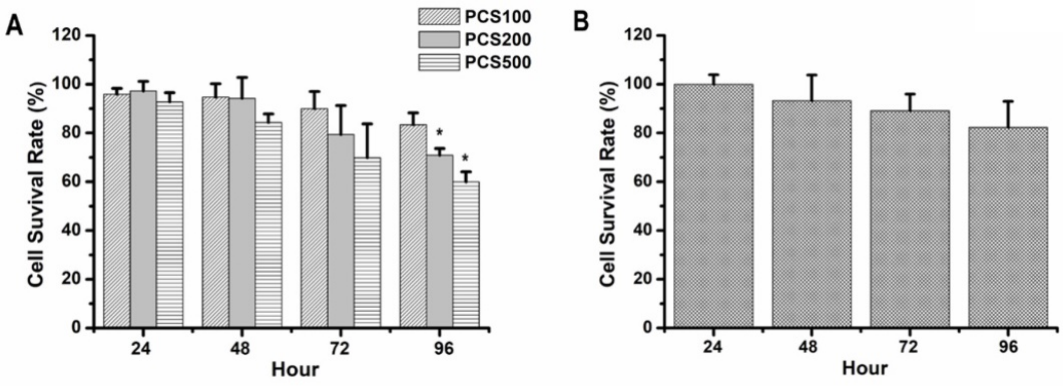
** Cell survival rate of HK-2 cells by PCS and curcumin treatments. (A)** Cells were treated with 100, 200, and 500 µM PCS. **(B)** Cells were treated with 8 µM curcumin. Cell survival rates were determined at 24-96 hours by MTT assay and calculated as A570 experimental group/A570 control group × 100%. The data were calculated from four independent experiments and presented as mean ± SD. *P < 0.05.

HK-2 cells, normal human renal proximal tubular epithelial cell line, were also treated with PCS. Our data showed that the survival rates were above 90% on 100 µM PCS-treated HK-2 cells during 24-96 hours and the survival rates were about 90 to 70% on 200 µM PCS-treated HK-2 cells during 24-96 hours (Fig. 2A). In addition, the survival rates were about 90 to 60% on 500 µM PCS-treated HK-2 cells during 24-96 hours (Fig. 2A). Our date indicated that PCS decreased cell survival rate of HK-2 cells with dose- and time-dependent manners. On the other hand, curcumin is a well-known anti-oxidative phytochemical. This study wanted to understand how does curcumin influence PCS-induced cytotoxic effects on HK-2 cells. Thus, the survival rate of HK-2 cell line was also determined with 8 µM curcumin treatment. Our data indicated that the survival rates were about 100-80% on HK-2 cells during 24-96 with curcumin treatments (Fig. 2B).

### Curcumin promoted PCS-induced cytotoxic effects on HK-2 cells

**Figure 3:**
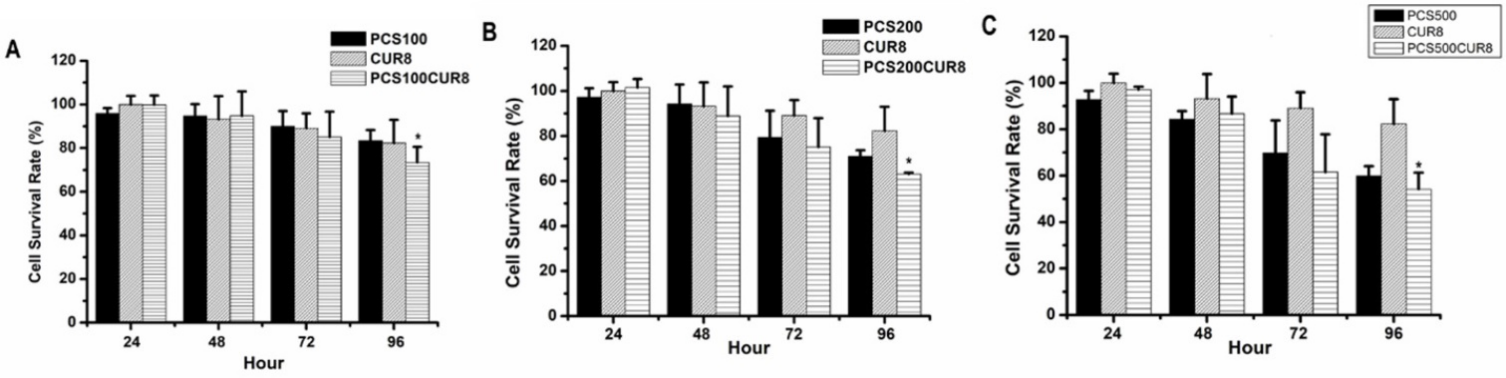
** Cell survival rate of HK-2 cells by PCS, curcumin, and PCS plus curcumin treatments. (A)** Cells were treated with 100µM PCS, 8 µM curcumin and 100µM PCS plus 8 µM curcumin. **(B)** Cells were treated with 200 µM PCS, 8 µM curcumin and 200 µM PCS plus 8 µM curcumin. **(C)** Cells were treated with 500 µM PCS, 8 µM curcumin and 500 µM PCS plus 8 µM curcumin. Cell survival rates were determined at 24-96 hours by MTT assay and calculated as A570 experimental group/A570 control group × 100%. The data were calculated from four independent experiments and presented as mean ± SD. *P < 0.05.

In order to study whether curcumin can influence PCS-induced cytotoxicity on HK-2 cells, combination treatments with PCS and curcumin on HK-2 cells were determine. The survival rates were above 80% on 100 µM PCS -treated and 8 µM curcumin treated-cells during 24-96 hour as well as the survival rates were also above 80% on 100 µM PCS -treated plus 8 µM curcumin treated-cells during 24-48 hour (Fig. 3A). However, the survival rate was under 80% on 100 µM PCS-treated plus 8 µM curcumin treated-cells at 96 hour (Fig. 3A). In addition, the survival rates were about 70% and 80% on 200 µM PCS-treated and 8 µM curcumin treated-cells at 96 hour respectively (Fig. 3B). However, the survival rate was about 60% on 200 µM PCS -treated plus 8 µM curcumin treated-cells at 96 hour (Fig. 3B). Observation on Figure 3C, the survival rate was under 60% only on 500 µM PCS -treated plus 8 µM curcumin treated-cells at 96 hour, however, the survival rates were above 60% on other groups. These results showed that combination treatments with PCS and curcumin may cause lower survival rates than only PCS or only curcumin treatment. That is curcumin promoted PCS-induced cytotoxic effects on HK-2 cells.

### PCS decrease G1/G0 phase percentage and caused S phase arrest on HK-2 cells

**Figure 4:**
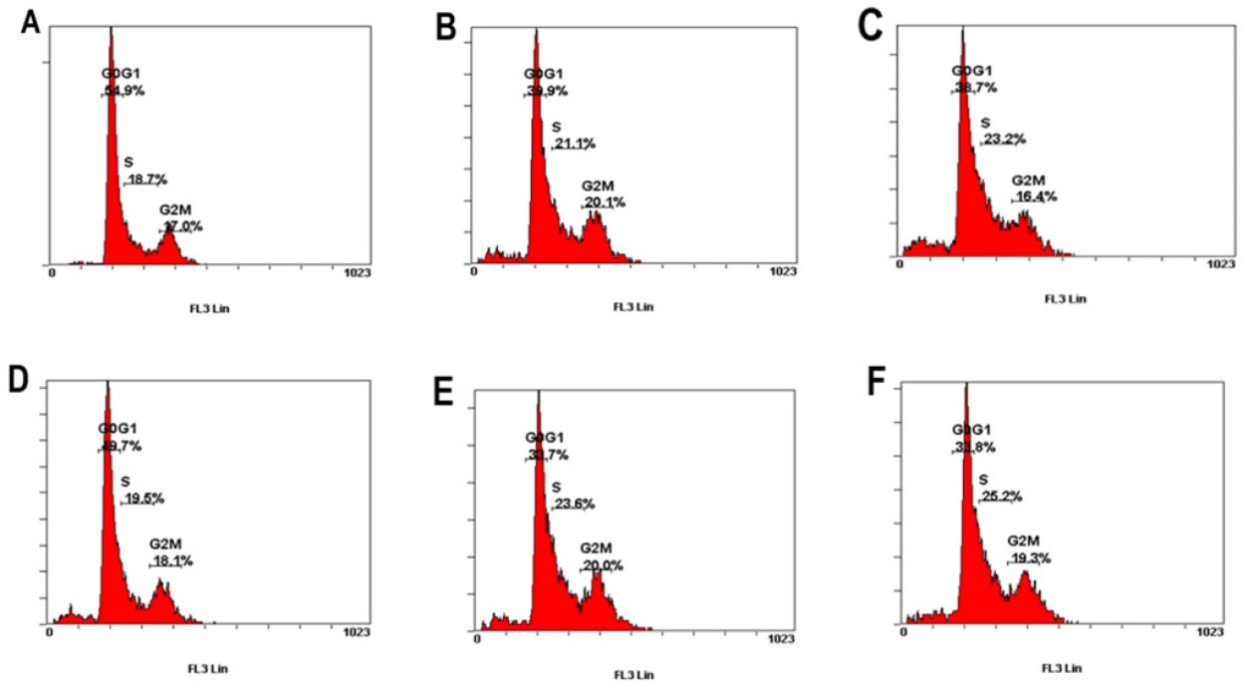
** The percentage of G0/G1, S, and G2/M phase was indicated on HK-2 cells. (A)** Control cells. **(B)** Cells were treated with 100µM PCS. **(C)** Cells were treated with 200 µM PCS. **(D)** Cells were treated with 8 µM curcumin. **(E)** Cells were treated with 100 µM PCS plus 8 µM curcumin. **(F)** Cells were treated with 200 µM PCS plus 8 µM curcumin.100 µM PCS. The cell cycle was analyzed by using flow cytometry at 48 hour.

This study further investigated whether PCS and curcumin can influence cell cycle on HK-2 cells. As shown in Figure 4 and Table 1, the percentage of G1/G0 was about 55% on control group and the percentages of G1/G0 was about 50% on curcumin-treated group. However, the percentages of G1/G0 were about 40%-34% on HK-2 cells with PCS and PCS plus curcumin treatments (Fig. 4 and Table 1). The data suggested PCS could decrease more G1/G0 percentage than curcumin treatment. In addition, the percentages of S-phase were about 18-19% on control and curcumin-treated groups while the percentages of S-phase were above 21-25% on PCS- and PCS plus curcumin-treated groups (Fig. 4 and Table 1). Our data indicated PCS can cause S-phase arrest on HK-2 cells.

### Sub-G1 levels were increased on HK-2 cells with PCS, curcumin, and PCS plus curcumin treatments

**Figure 5:**
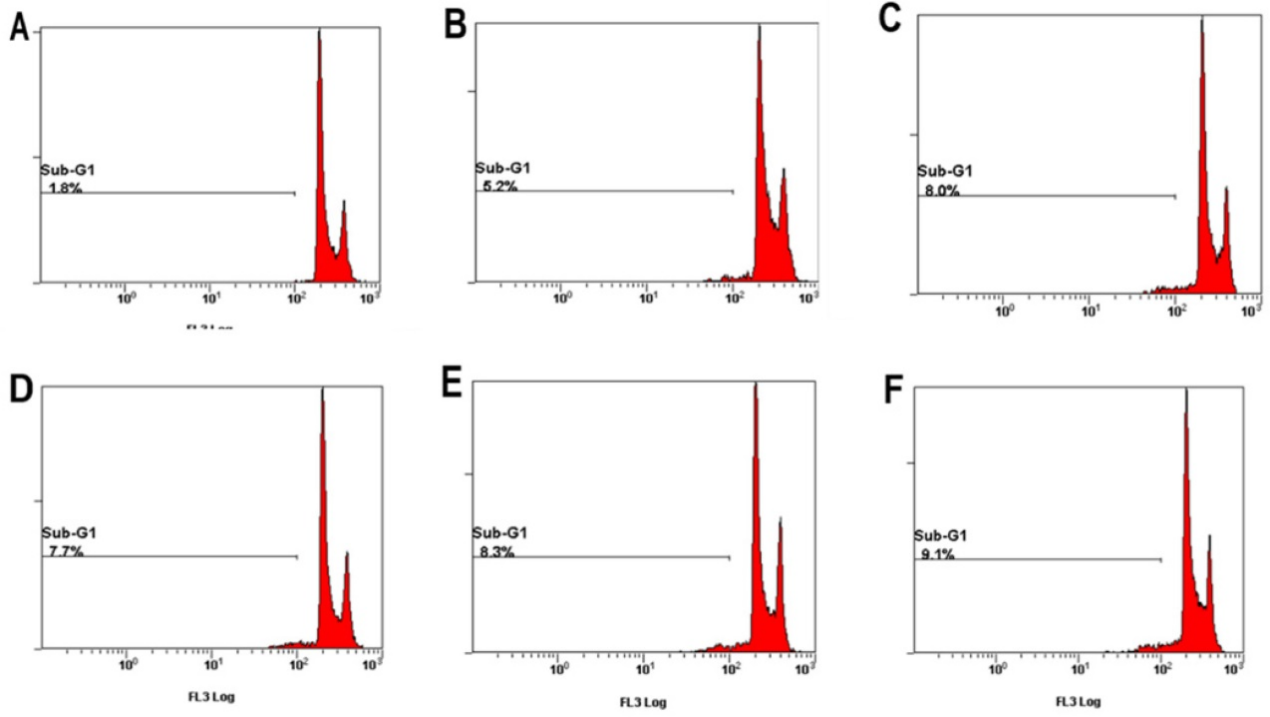
** The percentage of sub-G1 phase was indicated on HK-2 cells. (A)** Control cells. **(B)** Cells were treated with 100 µM PCS. **(C)** Cells were treated with 200µM PCS. **(D)** Cells were treated with 8 µM curcumin. **(E)** Cells were treated with 100µM PCS plus 8 µM curcumin. **(F)** Cells were treated with 200 µM PCS plus 8 µM curcumin.100 µM PCS. The cell cycle was analyzed by using flow cytometry at 48 hour.

Apoptotic cells can be determined in Sub-G1 phase with flow cytometry. As shown in Figure 5 and Table 1, the percentage of control group was 1.8% on control groups. However, the percentages of Sub-G1 phase were above 5-9% on PCS-, curcumin- and PCS plus curcumin-treated groups (Fig. 5 and Table 1). That is Sub-G1 levels were increased on HK-2 cells with PCS, curcumin, and PCS plus curcumin treatments. Our data indicated apoptotic death pathway may be involved on HK-2 cells with PCS and curcumin treatments.

### PCS and curcumin induced Caspase-3 activation and PARP cleavage on HK-2 cells

**Figure 6:**
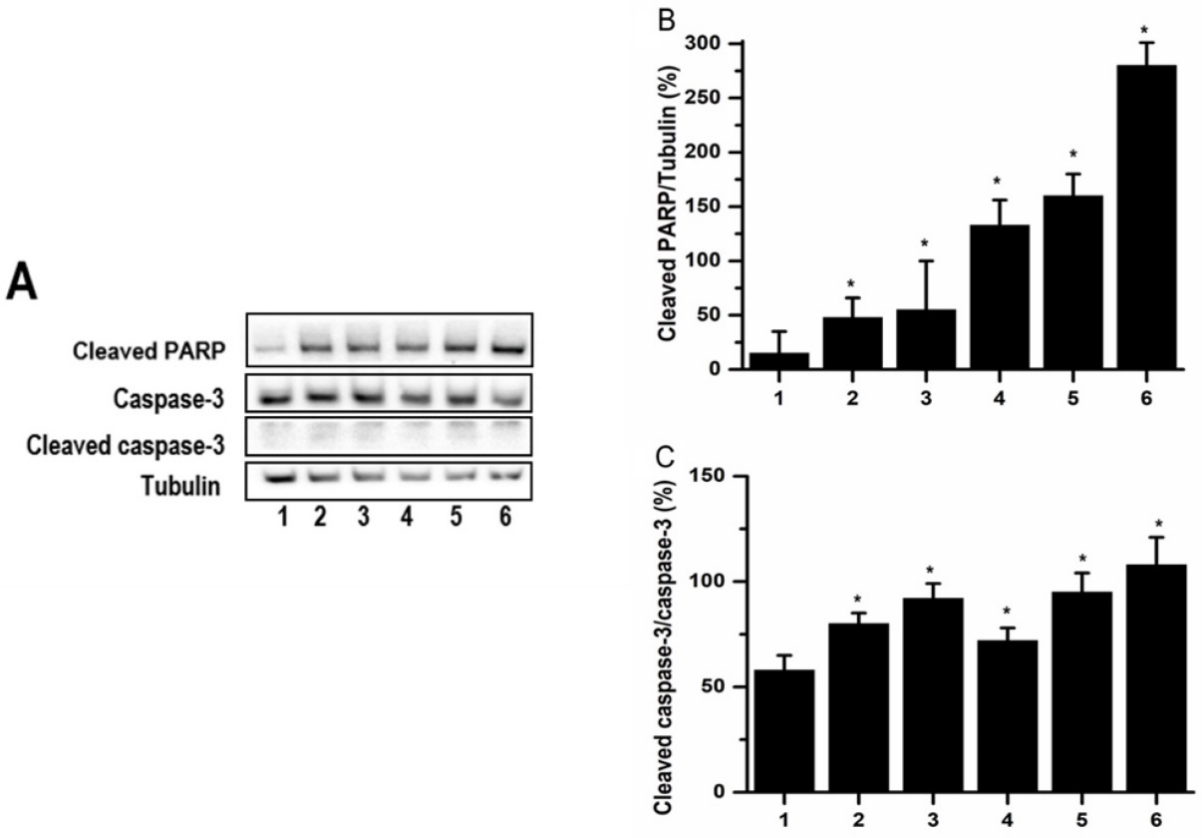
** The levels of caspase-3 and cleaved PARP. (A)** Caspase-3, cleaved caspase-3 and cleaved PARP were assayed by western blot. **(B)** Cleaved PARP/tubulin intensity ratio was calculated. **(C)** Cleaved caspase-3/caspase-3 intensity ratio was calculated. The proteins were determined after 48 hours treatments on control group (lane 1 and bar1), 100 µM PCS-treated group (lane 2 and bar 2), 100 µM PCS-treated group (lane 3 and bar 3), 200 µM PCS-treated group (lane3 and bar3), 8 µM curcumin-treated group (lane4 and bar 4), 100 µM PCS plus 8 µM curcumin-treated group (lane 5 and bar 5), 200 µM PCS plus 8 µM curcumin-treated group (lane6 and bar6). The data were determined from three independent experiments and presented as mean ± SD. *P < 0.05.

Apoptotic cell death may be induced through caspase-dependent or independent pathway. Casepase-3 and PARP were downstream of caspase pathways. In order to understand whether caspase-dependent death pathway was involved in PCS-caused apoptosis, the caspase-3 and cleaved PARP were determined by western blot. The experimental data showed that the levels of cleaved PARP were increased on PCS-, curcumin- and PCS plus curcumin-treated groups (Fig. 6A and 6B). Caspase-3 is an inactivated form existed into cells while cleaved caspase-3 is an activated form when caspase-3 pathway was induced. Observed on Figure 6, the levels of cleaved caspase-3 were increased on PCS-, curcumin- and PCS plus curcumin-treated groups as well as the ratios of cleaved caspase-3/caspase-3 were increased. That is, caspase-3 activation was induced by PCS, curcumin and PCS plus curcumin treatment. Our results indicated apoptotic death pathway through caspase-dependent was involved on PCS-, curcumin- and PCS plus curcumin-treated HK-2 cells.

## Discussion

Both Indoxyl sulfate (IS) and p-cresol sulfate (PCS) belong to protein-bound uremic toxins found extensively in chronic kidney disease (CKD) 10, 41-43, however, the cytotoxic effects on IS-treated cells were studied more than PCS-treated cells 21. Previous study indicated that PCS-induced fibrotic mechanism is similar to IS-induced 9. In addition, a study indicated PCS caused renal adverse effects of pig cells are similar to IS 21. However, the cytotoxic intensity between IS and PCS is not stated. Today our data showed that the cell survival rate of PCS-treated cells is lower than IS treated cells under the same dose conditions. Our study indicated PCS exerted a stronger cytotoxic intensity on renal tubular cells than IS. Thus, this study considered though both IS and PCS belong to protein-bound uremic toxins, PCS may cause a more serious injury than IS.

Previous studies suggested PCS induced renal cell injury progression may involve apoptosis, autophagy, and inflammation 22, 23. At present, our study showed PCS-induced cytotoxicity on human renal tubular cells were related to apoptosis and S-phase arrest. In addition, our data found that PCS reduce the G1/G0 phase percentage. The possible reasons of G1/G0 phase decreased may involve those the PCS accelerated cell cycle progression at G1/G0 phase and induced cells to enter the sub-G1 phase. In addition, cleaved PARP and caspase-3 activation were found on PCS-treated cells. Our study suggested the caspase-dependent apoptotic pathway was involved in PCS-induced cytotoxic effects on renal tubular cells.

Curcumin has anti-oxidant, anti-inflammatory and anti-apoptotic activities may be used on various diseases such as cardiovascular, and renal disease 44-47. In contract, curcumin also exerts cytotoxic effects on various cells 36, 37. Previous studies showed that curcumin caused cytotoxic effects on leukemia cells through autophagic, apoptotic and pathways and S phase arrest 35. In addition, a study showed that curcumin also caused autophagy and apoptosis on pancreatic cancer cells while curcumin can cause G2/M phase arrest but not S phase arrest on pancreatic cancer cells 37. Today our study found that curcumin could induce apoptosis and reduce G1 phase percentage. These studies indicated curcumin-induced cytotoxic effects may influence different cell cycle phase on different cell types.

**Figure 7:**
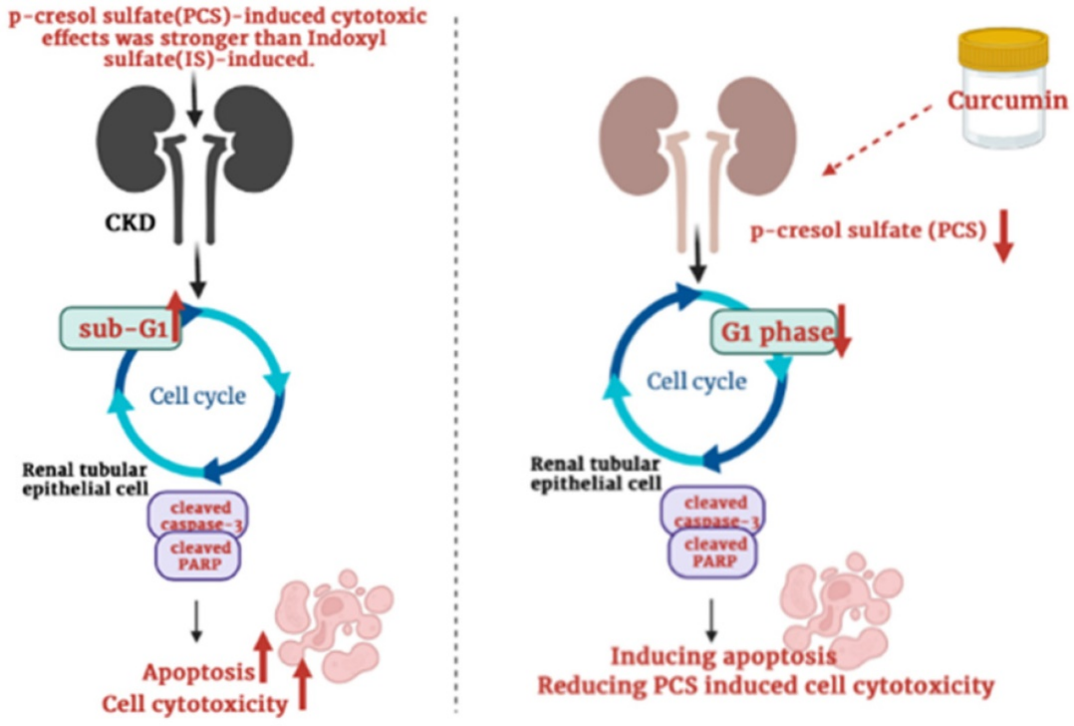
Schematic illustration depicting the roles of curcumin enhances p-cresyl sulfate-induced cytotoxic effects on renal tubular cells.

Though many studies showed that curcumin can protect cells against ischemia reperfusion-induced injury, cisplatin-induced nephrotoxicity, ferroptosis-mediated cell death, high glucose-induced EMT, lipopolysaccharide-induced renal inflammation, cyclosporine A‑induced fibrosis and gentamicin-induced nephrotoxicity 27-33, curcumin may also enhance cisplatin-induced cytotoxic effects 39. Up to now, whether curcumin can reduce or increase PCS-induced cytotoxic effects on renal cells was not reported. Our data firstly showed that curcumin might promote the cytotoxic degree on PCS-treated renal tubular cells. Due to PCS existed in CKD patients generally, our study suggested curcumin may be used carefully in clinical CKD (Fig. 7). In addition, our studies showed that curcumin could reduce G1 phase percentage and induce apoptosis, our studied considered curcumin could promote PCS-induced cytotoxic effects might be related to cell cycle alteration and apoptotic activation. Taken together, the study firstly demonstrated the PCS-induced cytotoxic effects were stronger than IS-induced, as well as curcumin could enhance PCS-induced cell cytotoxicity through caspase-dependent apoptotic pathway and cell cycle alteration. In addition, it is important curcumin would be used carefully in CKD patients.

## Figures and Tables

**Table 1:** 

		Control	PCS 100 µM	PCS 200 µM	Curcumin 8 µM	PCS100+CUR8	PCS200+CUR8
48 Hr	Sub-G	1.8%	5.2%	8%	7.7%	8.3%	9.1%
	G0/G1	54.9%	39.9%	38.7%	49.7%	33.7%	33.8%
	S	18.7%	21.1%	23.2%	19.5%	23.6%	25.2%
	G2M	17.0%	20.1%	16.4%	18.1%	20.0%	19.3%

The percentage of cell cycle phase was summarized at 48 hour on control cells, 100 µM PCS-treated cells, 200 µM PCS-treated cells, 8 µM curcumin-treated cells, 100 µM PCS plus 8 µM curcumin-treated cells and 200 µM PCS plus 8 µM curcumin-treated cells. The data was collected from Figures 4 and 5.
